# Response to induction chemotherapy as a prognostic indicator in locally advanced head and neck squamous cell carcinoma

**DOI:** 10.1007/s00432-024-06044-2

**Published:** 2024-12-04

**Authors:** Francesca Huwyler, Roland Giger, Ruben Bill, Daniel Rauch, Simon Haefliger

**Affiliations:** 1 Department of Medical Oncology, Inselspital, Bern University Hospital, University of Bern, Bern, Switzerland; 2 Department of Otorhinolaryngology, Head and Neck Surgery, Bern University Hospital, Inselspital, University of Bern, Bern, Switzerland

**Keywords:** Induction chemotherapy, Head and neck squamous cell carcinoma, Chemotherapy response, Survival Prediction

## Abstract

**Purpose:**

Induction chemotherapy (IC) for patients with locally advanced stage Head and Neck Squamous Cell Carcinomas (HNSCC) before radio-chemotherapy (RCT) or surgery remains a potential treatment option. This study analyzed how the response to IC correlates with survival outcomes.

**Methods:**

We conducted a retrospective single-center study at a tertiary cancer center. Tumors were categorized by anatomical site and response to IC (non-responders vs. responders). Data were analyzed using Kaplan-Meier survival curves and Cox regression analysis.

**Results:**

A total of 48 patients received IC. Of these, 33 patients were radiologically evaluable for response. The majority of evaluable patients received either TPF (Docetaxel, Cisplatin, 5-Fluorouracil) (58%) or TP (Docetaxel, Cisplatin) (24%) as their IC regimen. Tumor reduction of 30% or more was observed in 23 patients (69.7%), the tumor control rate was 97%. The 2-year event-free survival (EFS) in the IC evaluable population was 53.1%, overall survival (OS) was 63.6%, and recurrence-free survival (RFS) was 62.5%. Patients with laryngeal or hypopharyngeal tumors that did not respond to IC had a significantly poorer prognosis. This effect was not statistically significant in tumors of the oral cavity or oropharynx, where it was only observed as a trend.

**Conclusion:**

IC is highly effective in treating locally advanced stage HNSCC. The response to IC is prognostic for survival, particularly in cancers of the larynx and hypopharynx.

**Supplementary Information:**

The online version contains supplementary material available at 10.1007/s00432-024-06044-2.

## Introduction

Head and neck Squamous Cell Carcinoma (HNSCC) is one of the most prevalent cancers globally, with an annual incidence of approximately 770,000 cases and a mortality rate of 385,000 deaths in 2022 (*Cancer Today*, n.d.). The primary risk factors for HNSCC include tobacco and alcohol consumption, as well as viral infections such as human papillomavirus (HPV). Anatomically, these carcinomas are categorized into oral cavity, oropharyngeal, hypopharyngeal, and laryngeal carcinomas. Treatment strategies are determined by the disease stage and tumor location, with both surgery and radiotherapy (RT) offering curative potential in localized stages. For locally advanced tumors, a multimodal treatment comprising surgery, RT, and chemotherapy is typically employed (Johnson et al. 2020). Induction chemotherapy (IC), administered prior to surgery or RT aims to shrink tumors, reduce metastasis risk, and to enhance overall treatment outcomes, including survival rates.

The indication for IC in HNSCC remains controversial. Randomized trials have demonstrated that a three-drug combination of Cisplatin, Docetaxel, and 5-Fluorouracil (TPF) is most effective but associated with significant toxicity (Posner et al. 2007). Several randomized trials comparing IC plus radio-chemotherapy (RCT) versus RCT alone have not shown a statistically significant improvement in overall survival (OS) for the IC group (Cohen et al. 2014; Geoffrois et al. 2018; Ghi et al. 2017; Haddad et al. 2013a; Hitt et al. 2014). Consequently, RCT with Cisplatin alone remains the standard of care for patients with unresectable, locally advanced HNSCC. However, IC before RT remains crucial in the treatment of advanced laryngeal and hypopharyngeal carcinomas, where an organ-preserving approach is desired (Janoray et al., 2016). Despite the lack of significant improvement in OS, IC is still applied in various clinical scenarios, such as in patients with extensive, symptomatic tumors who must wait for the initiation of curative treatment with RCT or surgery. Additionally, IC is being explored to gain insights into tumor biology. Tumors resistant to chemotherapy are often also resistant to RT, likely due to shared pathways of resistance, such as those involved in DNA repair (Liu et al. 2021). The response to IC can thus help tailor subsequent treatments, whether RCT or surgery.

The aim of our study is to investigate how the response to IC has influenced long-term survival and recurrence rates in patients at our tertiary cancer center. Thereby, we aim to understand which patients can benefit from integrating IC into their treatment regimen.

## Methods

### Patients

We retrospectively analyzed patients with HNSCC who received IC at the University Hospital of Bern, Inselspital, Bern, Switzerland between 2005 and 2021. The inclusion criteria were as follows: adults > 18 years of age, treatment with IC and signed general consent for research. Patients treated before 2013 could be included without reconsent. Patients underwent radiographic assessments: Computed Tomography (CT), Magnetic Resonance Imaging (MRI) or Fluorodeoxyglucose Positron Emission Tomography (FDG-PET-CT) both before and after IC. Exclusion criteria: Patients who refused general consent for research after 2013 were excluded from the study. Additionally, we excluded patients with Epstein–Barr virus-positive nasopharyngeal carcinoma, sinonasal tumors and cancers of unknown primary in the head and neck region.

The study, including the reuse of health-related clinical data, received approval from the Cantonal Ethics Committee of Bern (Project-ID 2020–03074). The research was conducted in accordance with the Swiss Federal Human Research Act (HRA).

### Statistical analysis methods

Event free survival (EFS) was defined as the time from day 1 of IC to any of the following events: death, local, regional, or distant recurrence, local persistence after RCT, or secondary malignancy. Overall survival (OS) was defined as the time from day 1 of IC to death. Recurrence-free survival (RFS) was defined as the time from day 1 of IC to either local, regional, or distant recurrence, or local persistence after RCT. The maximal follow-up was limited to 5 years.

IC responders and non-responders: Tumor dimensions were extracted from radiographic reports and manually double-checked by the authors. In cases of uncertainty, the measurements were presented to trained radiologists for reassessment. However, a detailed re-assessment according to RECIST 1.1 criteria was not performed. Analogous to these criteria, we defined patients as ‘IC responders’ if their tumors demonstrated a reduction of 30% or more in the maximum diameter of the target lesion. Conversely, patients whose tumors did not achieve this level of reduction were categorized as IC non-responders’.

We conducted survival analysis using R and RStudio. Specifically, we employed the R packages: survivalAnalysis (Therneau 2024) and survminer (Drawing Survival Curves Using “ggplot2” [R Package Survminer Version 0.4.9], 2021) for Kaplan-Meier survival curves and Cox regression analysis. Figures were created using these packages and subsequently arranged for publication using Affinity Publisher 2.

## Results

**Fig. 1:**
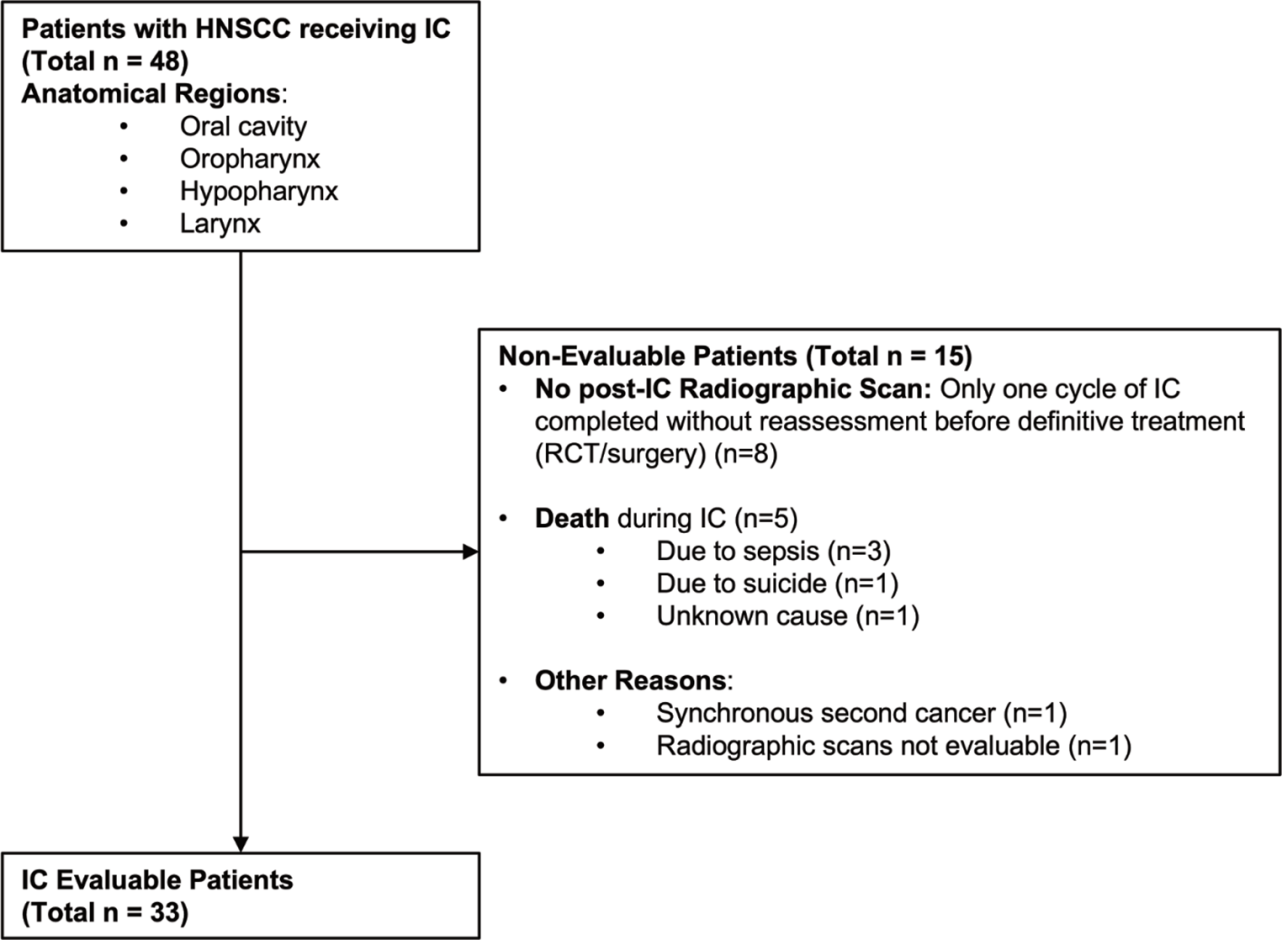
Study flow chart. IC: Induction chemotherapy, RCT: Radio-chemotherapy

In this retrospective cohort study, we identified 48 patients who received IC for HNSCC, involving anatomical sites such as the oral cavity, oropharynx, larynx, and hypopharynx. Of these, 33 patients underwent radiologic tumor assessment post-IC and were thus evaluable for tumor response. Fifteen patients were not evaluable for response (Fig. 1), primarily because they received only one cycle of IC before proceeding directly to definitive treatments such as RCT or surgery. There were five recorded deaths, three of which were due to IC-related neutropenic sepsis. The median follow-up time was 3.6 years for the entire cohort and 4.2 years for the IC evaluable population.

### Patients’ characteristics

The baseline characteristics of the IC evaluable patients are shown in Table 1. Notably, fewer patients with oral cavity cancers were included. The majority of patients were male. Most patients received either the triplet chemotherapy regimen with Docetaxel, Cisplatin, and 5-Fluorouracil (TPF) or the double combination with Docetaxel and Cisplatin (TP). The median number of administered IC cycles was 3. Following IC, all but two patients received RCT. Due to the absence of routine HPV testing prior to 2017, HPV status was reported in only a minority of cases. Most patients presented with locally advanced tumors, characterized by higher T and N stages. Notably, seven patients were staged as having metastatic disease. Of these, six had small pulmonary lesions detected on FDG-PET-CT scans, whose nature was undetermined at the time of primary diagnosis. One patient had a histologically confirmed single liver metastasis, which was subsequently treated with radiosurgery. There are no statistically significant differences in patient characteristics between the groups of responders and non-responders to IC.

**Table 1:** Patients’ characteristics

Characteristics		All				Non-responders				Responders			
Total (n)		33				10				23			
Age (median)		62	(42–81)			65	(49–81)			57	(42–75)		
Anatomical site (n)	Oral cavity	4		12	%	2		20	%	2		8.7	%
	Oropharynx	11		33	%	4		40	%	7		30	%
	Hypopharynx	8		24	%	0		0	%	8		35	%
	Larynx	10		30	%	4		40	%	6		26	%
Sex (n)	male	27		82	%	8		80	%	19		83	%
	female	6		18	%	2		20	%	4		17	%
Induction chemotherapy (n)	TPF	19		58	%	6		60	%	13		57	%
	TP	8		24	%	2		20	%	6		26	%
	CarboTF	3		9.1	%	1		10	%	2		8.7	%
	PF	1		3	%	0		0	%	1		4.3	%
	CarboF	1		3	%	1		10	%	0		0	%
	TPF > CarboTF	1		3	%	0		0	%	1		4.3	%
Number of cylces (median)		3	(1–5)			3	(1–5)			3	(2–4)		
Following treatment (n)	RCT	31		94	%	9		90	%	22		96	%
	surgery	1		3	%	0		0	%	1		4.3	%
	surgery + adj RCT	1		3	%	1		10	%	0		0	%
HPV status (n)	positive	3		9.1	%	0		0	%	3		13	%
	negative	9		27	%	3		30	%	6		26	%
	NA	21		64	%	7		70	%	14		61	%
T (n)	T1-2	6		18	%	1		10	%	5		22	%
	T3-4	27		82	%	9		90	%	18		78	%
N (n)	N0-1	10		30	%	2		20	%	8		35	%
	N2-3	23		70	%	8		80	%	15		65	%
M (n)	M0	26		79	%	8		80	%	18		78	%
	M1	7		21	%	2		20	%	5		22	%

RCT - Radiochemotherapy; HPV - Human Papillomavirus; TPF - Docetaxel, Cisplatin, 5-Fluorouracil; TP - Docetaxel, Cisplatin; CarboTF - Carboplatin, Docetaxel, 5-Fluorouracil; PF - Cisplatin, 5-Fluorouracil; CarboF - Carboplatin, 5-Fluorouracil; adj - adjuvant; There is no statistically significant difference between non-responders and responders (*p* > 0.05; Mann-Whitney U test or Chi-squared test, respectively)

### Response rate

**Fig. 2:**
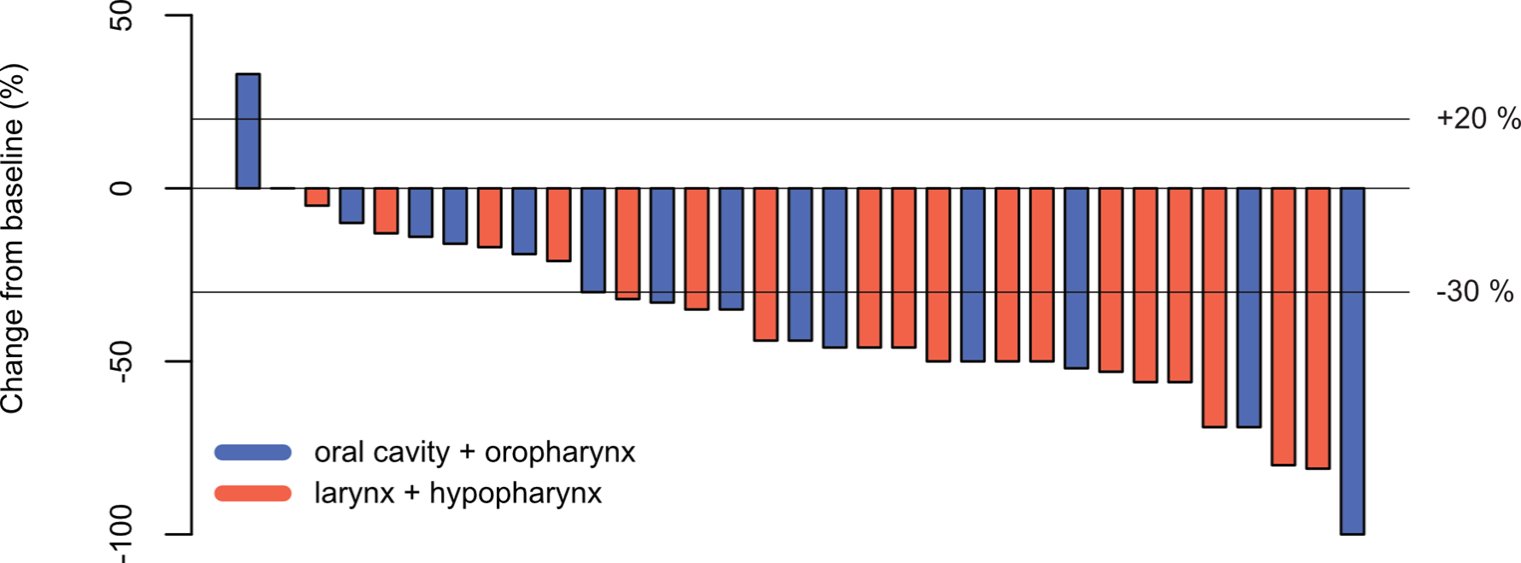
Waterfall plot of response after induction chemotherapy based on longest diameter of target lesions (%). Patients exhibiting a tumor reduction of 30% or more are categorized as responders, while those with less than 30% reduction or disease progression are categorized as non-responders

Among the 33 IC evaluable patients in our study, 23 demonstrated a response, characterized by tumor reduction of 30% or more. Additionally, 9 patients exhibited stable disease, while 1 patient experienced tumor progression. This results in a response rate of 69.7% (23 out of 33) and a tumor control rate of 97% (32 out of 33) (Fig. 2).

As mentioned before we have limited information about the HPV status. Therefore, the subgroup of patients with known HPV status (*n* = 12) is too small for detailed statistical analyses. However, all patients with positive HPV status (*n* = 3) were responders to IC, including the patient who achieved a complete response.

### Outcome

In the total population, median survival was 15.3 months (95% confidence interval (CI) 8.7–51.5) for EFS and 49.9 months (95% CI 19.7– NA) for OS (Supplemental Fig. S1). In the IC evaluable population, median survival was 24.4 months (95% CI 9.2 - NA) for EFS, 56.2 months (95% CI 21.2 - NA) for OS, and 52.4 months (95% CI 15.6 - NA) for RFS (Supplemental Fig. S2). Most events occurred within the first 12 months after the start of IC. Focusing on the IC evaluable cohort, five patients showed local tumor persistence following RCT. The 2-year survival data shows EFS at 53.1%, OS at 63.6% and RFS at 62.5%. These numbers closely align with previous data from randomized phase 3 trials, such as the TAX 324 study, which demonstrated a 2-year PFS of 53% and OS of 67% in the TPF group (Posner et al. 2007). This suggests that our cohort is representative for studying IC in HNSCC.

**Fig. 3:**
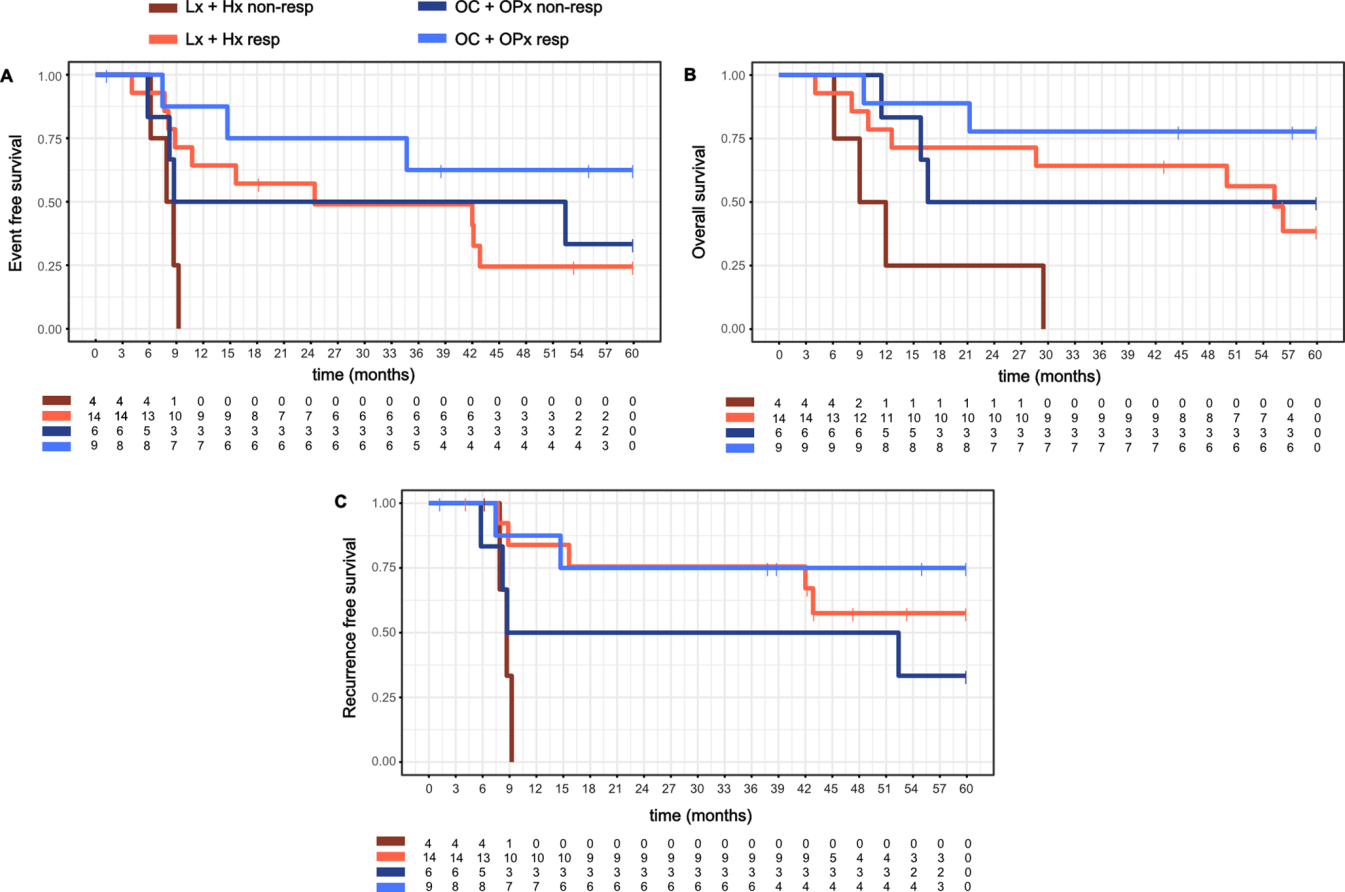
Kaplan-Meier estimates of survival grouped according to anatomical sites. Upper ENT group includes tumors located in the oral cavity and oropharynx, while the lower ENT group comprises tumors in the larynx and hypopharynx. The plots illustrate Event free survival (**A**), Overall survival (**B**), and Recurrence free survival (**C**). The overall log-rank test p-values are for (**A**) *p* = 0.015, (**B**) *p* = 0.009, (**C**) *p* = 0.024. Lx + Hx non-resp - Larynx + Hypopharynx Cancers not responding to Induction Chemotherapy. Lx + Hx resp - Larynx + Hypopharynx Cancers responding to Induction Chemotherapy. OC + OPx non-resp - Oral cavity + Oropharynx Cancers not responding to Induction Chemotherapy. OC + OPx resp - Oral cavity + Oropharynx Cancers responding to Induction Chemotherapy

**Fig. 4:**
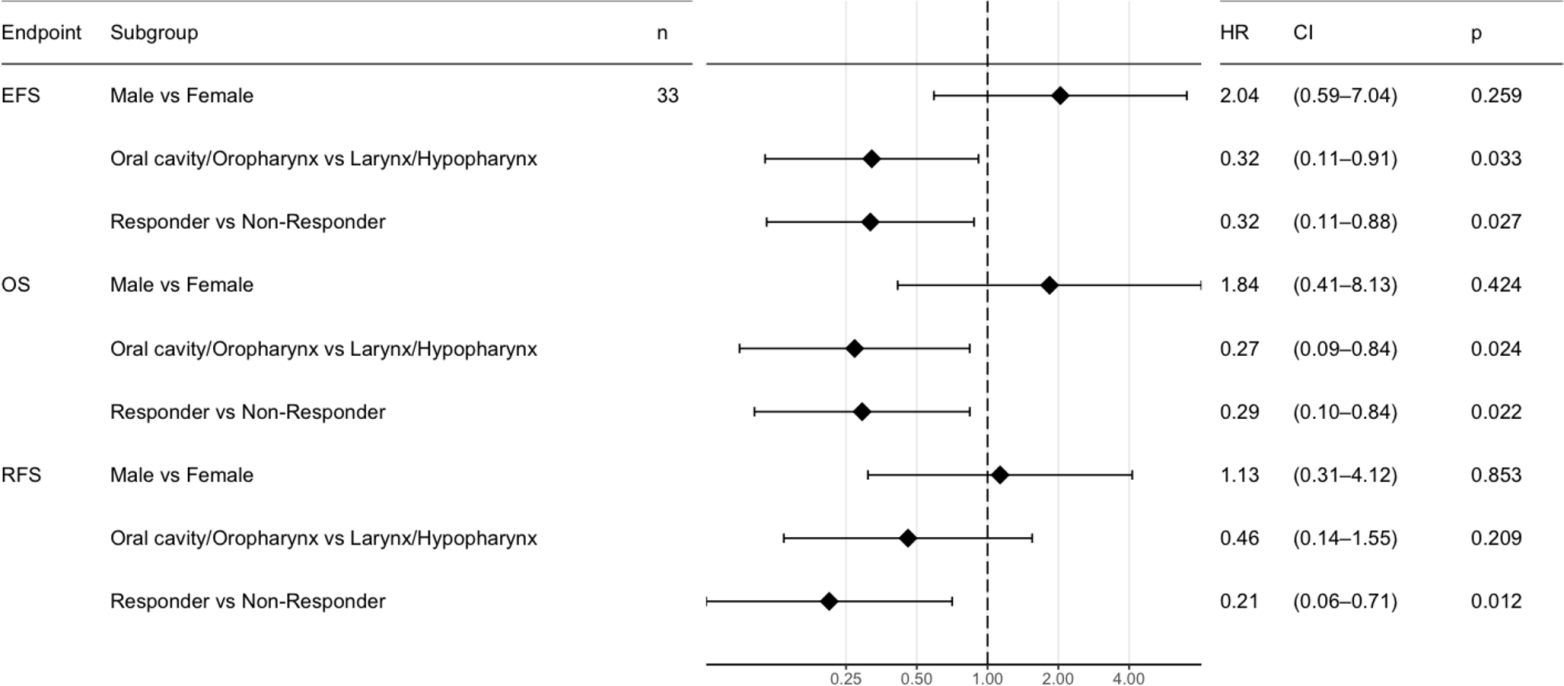
Multivariate analysis of subgroups. Cox Regression analysis using the covariates sex, anatomical site and response to induction chemotherapy. EFS - Event free survival, OS - Overall survival, RFS - Recurrence free survival, HR - Hazard Ratios, CI - Confidence interval

Next, we categorized the tumors into anatomical groups: oral cavity and oropharynx - upper head and neck (HN) region-, or larynx and hypopharynx - lower HN region. Our analysis revealed that non-responsive tumors in the lower HN region have the poorest outcomes (Fig. 3). Similarly, non-responsive tumors in the upper HN region exhibit a trend towards worse outcomes in comparison to responsive tumors, although this difference is not statistically significant in pairwise comparisons. Interestingly, non-responsive tumors in the upper HN region have a prognosis comparable to that of responsive tumors in the lower HN region. The multivariate analysis shows that the subgroup of upper HN tumors has generally a better outcome in terms of EFS and OS (Fig. 4). The subgroup with responding tumors has a better outcome regarding all endpoints. These results indicate that response to IC is prognostic for survival, particularly in the lower HN region.

## Discussion

The effect of IC has already been investigated in various randomized clinical trials. In our cohort, we aimed not to investigate IC itself, but rather to determine the extent to which the response to IC can predict outcomes in a real-world patient cohort. Our findings indicate that patients with tumors in the lower HN region who do not respond to IC have poor long-term survival. Conversely, patients who do respond to IC and receive an organ-preserving therapy concept involving RCT have a high chance of achieving long-term tumor control. For upper HN tumors we observe a similar but statistically not significant trend. The correlation between IC response and survival outcomes has been previously established in a meta-analysis (Kiong et al. 2018). However, this analysis did not provide a breakdown of the data according to individual anatomical subgroups. Additionally, only three studies in the meta-analysis provided long-term data with five years of follow-up. A retrospective study in an Asian cohort also confirms the favorable overall survival in IC responders (Lee et al. 2022). Our study supports these findings in a Caucasian patient cohort. The study further suggests that IC non-responders may benefit more from subsequent surgery rather than RCT. Since the majority of patients in our cohort were treated with RCT after IC, we cannot address the question of follow-up therapy. However, the patient with the longest OS in the subgroup of non-responsive lower HN tumors underwent surgery followed by adjuvant RCT after IC. Another Asian study analyzed patients with oropharyngeal carcinoma who received IC focusing on the difference between HPV positive and negative tumors (Zhang et al. 2023). HPV-positive tumors responded better to IC, and the response to IC was associated with better OS and progression-free survival (PFS) in both HPV-positive and HPV-negative patients. In our cohort, all HPV-positive tumors responded to IC.

It has been shown that a delay of more than 7 weeks between diagnosis and the start of curative treatment negatively impacts OS (Murphy et al. 2016). If the initiation of RCT is delayed due to clinical or administrative reasons, bridging chemotherapy can be used to control tumor progression. Our data can support this approach: The tumor control rate in our cohort, as well as in comparable cohorts, is very high. However, due to potentially fatal side effects such as febrile neutropenia (11–23%), supportive therapy with prophylactic G-CSF and close clinical monitoring is recommended (Haddad et al. 2013b).

Clinical trials are currently investigating the combination of immune checkpoint inhibitors with chemotherapy in neoadjuvant therapy concepts (Zhao et al. 2024). It will be interesting to see to what extent these findings can be applied to new induction therapy concepts.

Our study has several limitations. It is a single-center retrospective study with a relatively small number of patients. Tumor extent was assessed using various radiographic techniques (FDG-PET-CT, MRI, CT). However, by broadly categorizing tumor response into ‘IC responders’ and ‘IC non-responders’, we aimed to minimize measurement errors. To be included in the final analysis, patients were required to undergo two radiologic examinations—one before and one after IC. This criterion excluded patients who received only one IC cycle and proceeded directly to definitive treatment, as well as those who died during the IC. Additionally, we excluded tumors of the nasal cavity, paranasal sinuses, and nasopharynx from the analysis, therefore our findings cannot be applied to these entities.

In summary, our retrospective real-world dataset demonstrates that patients who respond to IC have a favorable prognosis in terms of survival, particularly for tumors of the larynx and hypopharynx. The response to IC can be a valuable indicator for selecting appropriate further treatment modalities.

## Electronic Supplementary Material

Below is the link to the electronic supplementary material.

Supplementary Material 1

## Data Availability

No datasets were generated or analysed during the current study.
