# Supplementary material for: Response to induction chemotherapy as a prognostic indicator in locally advanced head and neck squamous cell carcinoma
Source: J Cancer Res Clin Oncol. 2024 Dec 4;151(1):2. doi: 10.1007/s00432-024-06044-2 (PMC11615028; doi:10.1007/s00432-024-06044-2)
Supplement: Supplementary file 1 — Supplementary Material 1 [file 432_2024_6044_MOESM1_ESM.docx]

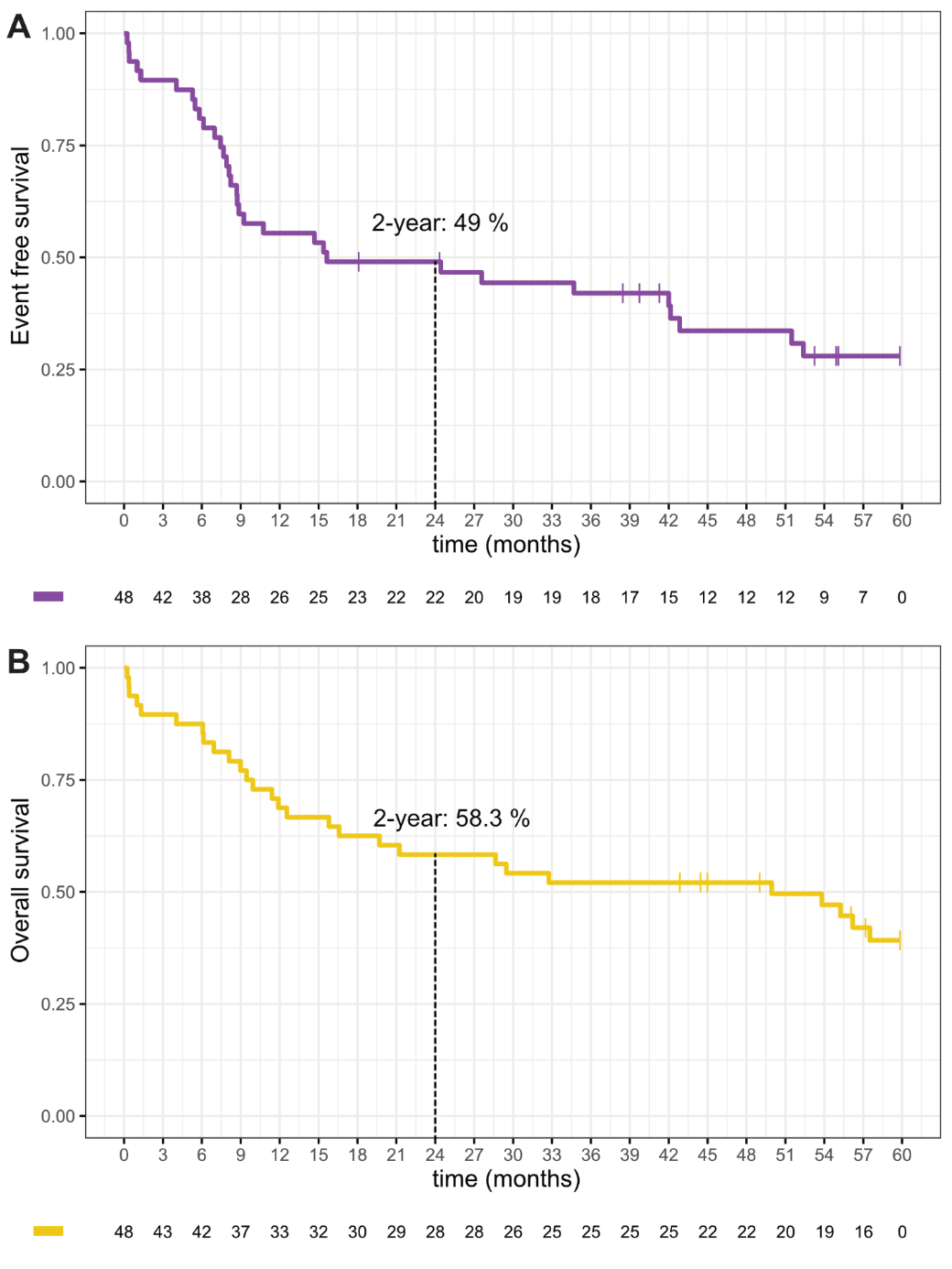


**Figure S1. Kaplan-Meier estimates of survival in the total population (n = 48).** Event free survival (A), Overall survival (B)

**
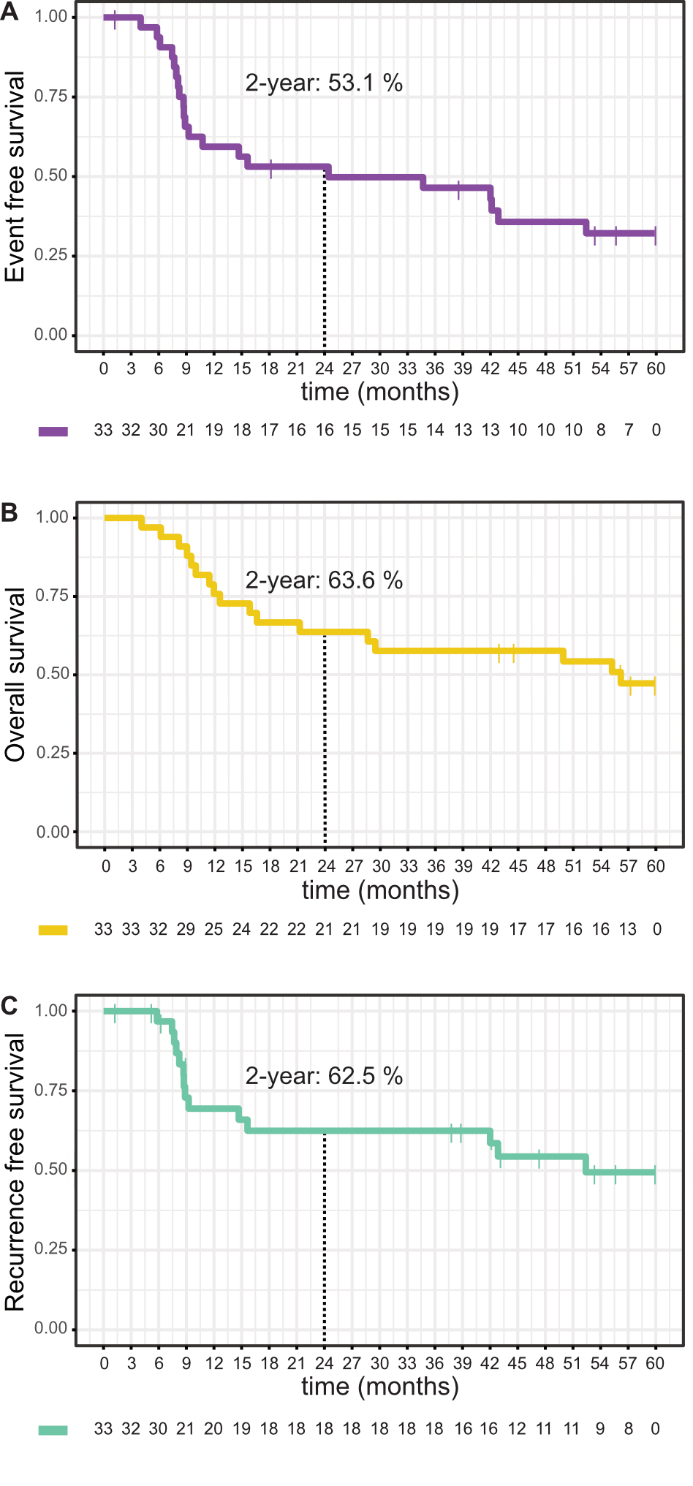
**

**Figure S2. Kaplan-Meier estimates of survival in the IC evaluable cohort (n = 33).** Event free survival (A), Overall survival (B), Recurrence free survival (C)
